# Advanced Simulation of Removing Chromium from a Synthetic Wastewater by Rhamnolipidic Bioflotation Using Hybrid Neural Networks with Metaheuristic Algorithms

**DOI:** 10.3390/ma14112880

**Published:** 2021-05-27

**Authors:** Hamid Khoshdast, Alireza Gholami, Ahmad Hassanzadeh, Tomasz Niedoba, Agnieszka Surowiak

**Affiliations:** 1 Department of Mining Engineering, Higher Education Complex of Zarand, Zarand 7761156391, Iran; 2 Department of Mineral Processing, Tarbiat Modares University, Tehran 14115-111, Iran; ar.gholami2744@gmail.com; 3 Independent Scholar, Am Apostelhof 7A, 50226 Frechen, Germany; a.hassanzadeh@gmx.de; 4 Department of Geoscience and Petroleum, Faculty of Engineering, Norwegian University of Science and Technology, 7491 Trondheim, Norway; 5 Department of Environmental Engineering, Faculty of Mining and Geoengineering, AGH University of Science and Technology, al. Mickiewicza 30, 30-059 Krakow, Poland; asur@agh.edu.pl

**Keywords:** wastewater treatment, rhamnolipidic bioflotation, kinetics, hybrid neural network, metaheuristic algorithms

## Abstract

This work aims at presenting an advanced simulation approach for a novel rhamnolipidic-based bioflotation process to remove chromium from wastewater. For this purpose, the significance of key influential operating variables including initial solution pH (2, 4, 6, 8, 10 and 12), rhamnolipid to chromium ratio (RL:Cr = 0.010, 0.025, 0.050, 0.075 and 0.100), reductant (Fe) to chromium ratio (Fe:Cr of 0.5, 1.0, 1.5, 2.0, 2.5, 3.0), and air flowrate (50, 100, 150, 200 and 250 mL/min) were investigated and evaluated using Analysis of Variance (ANOVA) method. The RL as both collector and frother was produced using a pure strain of Pseudomonas aeruginosa MA01 under specific conditions. The bioflotation tests were carried out within a bubbly regimed column cell with the dimensions of 60 × 5.70 × 0.1 cm. Four optimization techniques based on Artificial Neural Network (ANN) including Cuckoo, genetic, firefly and biogeography-based optimization algorithms were applied to 113 experiments to identify the optimum values of studied factors. The ANOVA results revealed that all four variables influence the bioflotation performance through a non-linear trend. Their influences, except for aeration rate, were found statistically significant (*p*-value < 0.05), and all parameters followed the normal distribution according to Anderson-Darlin (AD) criterion. Maximum chromium removal of about 98% was achieved at pH of 6, rhamnolipid to chromium ratio of 0.05, air flowrate of 150 mL/min, and Fe to Cr ratio of 1.0. Flotation kinetics study indicated that chromium bioflotation follows the first-order kinetic model with a rate of 0.023 sec^−1^. According to the statistical assessment of the model accuracy, the firefly algorithm (FFA) with a structure of 4-9-1 yielded the highest level of reliability with the mean squared, root mean squared, percentage errors and correlation coefficient values of test-data of 0.0038, 0.0617, 3.08% and 96.92%, respectively. These values were evidences of the consistency of the well-structured ANN method to simulate the process.

## 1. Introduction

Since hexavalent chromium is highly toxic, its effluent in electroplating, tannery, dyes and pigments, film and photography, and mining industries is severely hazardous for the environment and creates serious damage to human and animal bodies [1]. The highest Cr(III) and Cr (VI) amounts allowed in wastewater are 5 mg/L and 0.05 mg/L, respectively [2]. Several treatment techniques have been developed to remove chromium pollutants from wastewaters including chemical precipitation, coagulation-flocculation, electrocoagulation, membrane filtration, ion flotation, ion exchange, activated carbon and adsorption [3,4,5]. Among these, merging biotechnology with conventional treatment techniques has been of great interest in recent decades. However, there is considerable lack of information about the biotreatment and biosurfactants on removing Cr (VI). Further, the impact of effective operating parameters has not been adequately explored in the literature yet. 

Nowadays, these microbial products, often called bio-surfactants, have been found as promising substitutes of petroleum-derived surfactants widely used in processing and treatment industries. The chief advantages of such bioproducts in comparison to synthetic surfactants are their high stability at various pHs and temperatures as well as their considerable compatibility with the environment due to their appreciable biodegradability and low toxic effects. Moreover, recent advances in their production, especially from renewable resources, have opened up promising horizons for their large-scale applications compared to their chemical competitors [6,7]. In this context, rhamnolipids have acted as a surface-active agent consisting of one (mono-RL) or two (di-RL) (L)-rhamnose groups connected to the hydrophilic group composed of one or two β-hydroxy fatty acids through a glycoside bond [8,9]. Its surface forces and foamabilities were studied in detail by Khoshdast et al. [10]. They showed that rhamnolipid has strong static and dynamic frothability indices. Moreover, rhamnolipid reveals low selectivity in three-phase flotation systems in comparison with conventional alcoholic and etheric frothers. Renfro et al. [11] studied the equilibrium and kinetic adsorptions of rhamnolipid in soil system and showed that the thermodynamic characteristics of the surface of rhamnolipid molecules can significantly control the rate of rhamnolipid transport. In addition, formation of rhamnolipid micelles is dramatically influenced by the attractive forces between RL molecules. Investigation of the surface tension related properties of pure rhamnolipids at natural pH in absence or in presence of mineral additives indicated that with the use of electrolytes, some structural changes and interactions among hydrophilic and hydrophobic groups may take place through different types of forces such as electrostatic and hydrogen interactions [12]. Zhao et al. [13] conducted comparative studies on the structural composition and surface/interface activity rhamnolipids using hydrophobic or hydrophilic substrates and interestingly showed that the type of substrate used for bacterial cultivation significantly affects the number of homologues in RL structure and its surface activity. Rekiel et al. [14] evaluated the adsorption properties of rhamnolipid and ethanol in water/ethanol solutions and showed that ethanol/RL mixture had no synergetic effect on the surface tension of the aqueous solution. Recently, Ahmad et al. [15] investigated the stability and efficiency of RL biosurfactants under extreme conditions and indicated that mono-rhamnolipids biosurfactant can be stable at 60 °C, pH 10 and 10% salinity.

Due to the high binding capacity with metal ions, some researchers have investigated the rhamnolipid-facilitated treatment of soils and waters contaminated by chromium ions. For example, Juwarkar et al. [16] assessed the potential of using the di-rhamnolipid biosurfactant as an ion collector to remove cations from multi-metal contaminated soil. They showed that within 36 hours of leaching study, di-rhamnolipid can provide Cr removal (92%) from the contaminated soil 13-fold higher than other heavy metals. The studied soil samples varied in pH and Cr contents from 6.4 to 7.8 and 50 to 940 ppm, respectively. Removal and reduction of chromium by rhamnolipid were also investigated by Ozturk et al. [17]. They showed that the removal efficiency of chromium directly depended on the biosurfactant concentration. Moreover, the presence of RL bacterial strain can appropriately reduce Cr(VI) to Cr(III). Abbasi-Garravand and Mulligan [18] coupled the micellar enhanced ultrafiltration and reduction techniques to remove Cr(III) and Cr(VI) ions from an aqueous solution. They examined initial concentration of hexavalent chromium (10–400 ppm), pH (6–10), and rhamnolipid concentration (0.1–2 %vol.) to assess the effect of these operating variables on the reduction of Cr(VI). They found that the maximum Cr(VI) reduction efficiency (98.7%) could be achieved at a Cr(VI) concentration of 10 mg/L, RL concentration of 2% (vol/vol.) and initial solution pH of 6. Chen et al. [19] employed rhamnolipid as the washing agent to remove chromium and some other heavy metals in river sediment. They investigated the effects of rhamnolipid concentration (0.2–3 %wt.), washing time (0.5–40 h), solution pH (3–11), and liquid/solid ratio (5–20). They reported that heavy metal washing was favoured (47.85% of Cr removal) at an RL concentration of 0.8% after 12 h at pH 7.0. Shojaei and Khoshdast [20] investigated the potential treatment of wastewater polluted by chromium using precipitate flotation in the presence of rhamnolipid biosurfactants. They evaluated the effect of solution pH (5–8), Rl concentration (1–10 ppm), aeration rate (50–200 ml/min) and precipitant portion (1.5–3). They showed that optimal removal of 96.75% could be achieved at pH 8, aeration rate of 50 ml/min, and rhamnolipid and co-precipitant to metal ratios of 0.01 and 3, respectively. It was also found that the presence of cations and anions can reduce the process efficiency. These findings were in good agreement with those reported by Abyaneh and Fazaelipoor [21] but with lower Cr removal (96.1%). Recently, Yang et al. [22] studied the adsorption of some heavy metals including Cr(VI) on some clays such as ferrihydrite and nontronite, and the impacts of RL biosurfactant on desorption efficiency. Their results revealed that Cr(VI) was adsorbed on ferrihydrite and goethite with better adsorption rates at pH 8.0, and its maximum adsorption ranged from 58.8% to 90.7%. Moreover, rhamnolipids were found to be effective for improving desorption of cationic contaminants from the surface of minerals using a bio-washing process.

Ion flotation is a promising process, in which cationic species are removed from aqueous solution using an appropriate collector. However, despite the relatively simple mechanical and physical aspects, the flotation process is very complicated mechanically. Such complexities mainly come from the interaction between different reagents and phases involved in the system. Therefore, modelling and simulation of flotation processes has been always a challenging issue of debate. A recent approach to simulate complicated separation techniques is to use expert system methods such as artificial neural networks. These networks are among the most widely used intelligent algorithms that transmit the knowledge or the rule behind data into a network structure by processing experimental data. ANN can be utilized to implement difficult functions in numerous areas, such as pattern recognition, visual system, classification and controlling systems. Nowadays, problems that are difficult for humans or ordinary computers can be solved by properly training neural networks [23]. One of the main applications of neural networks is forecasting based on a set of input data that has also yielded excellent results. Thanks to their good performance, ANNs have been frequently used in various scientific fields, including mining and mineral processing [24,25,26,27,28,29,30].

In this work, the removal of chromium from synthetic wastewater using rhamnolipidic flotation has been studied. The optimization, kinetics, and intelligent simulation of the flotation process were also investigated using a set of advanced expert algorithms coupled with experimental results. To the best of the author’s knowledge, such an advanced simulation of similar processes has not yet been addressed in the open literature.

## 2. Materials and Methods

### 2.1. Biosurfactant Production and Reagents 

The biosurfactant production was performed using a pure strain of *Pseudomonas aeruginosa* MA01. The bacteria’s pre-culture was undertaken on nutrient broth over a night at 30 °C and 200 rpm following inoculation into an optimum medium for batch fermentation to produce the bacterial product. A modified culture medium was prepared by adding 10 mL of glycerol as carbon source and appropriate amounts of nutrients to distilled water and was incubated at 37 °C and 150 rpm for 24 h [31]. The ingredients and consumption dosage of culture medium is listed in Table 1. Afterwards, the cell-free supernatant (CFS) was prepared by centrifugation of the cell suspension at the rate of 15,000 × g for 20 min at 4 °C. pH of the CFS was subsequently reduced to 2 with 1 M hydrochloric acid and rested for 24 h in a cool place to precipitate the rhamnolipid product. The precipitated RL was later separated via centrifuging at 15,000× *g* for 60 min. Then, the settled biomass was washed out by acidic solution following centrifugation at 15,000× *g* for 20 min. After mixing the obtained RL product with ethyl acetate of equal volume, shaking for 10 min, and centrifuging at 15,000× *g* for 20 min, the acetate phase was removed by evaporation at 40 °C using a vacuum rotary evaporator [10]. Finally, about 6 g of viscous light brown RL product was obtained per liter of the medium. Detailed information regarding the produced RL’s properties, its structure and frothability features can be found in our previous works [10,31,32]. It is well demonstrated that RL biosurfactants have significant foaming characteristics. Compared to frothing reagents conventionally used in the flotation processes, such as Dowfroth (DF-250) and methyl isobutyl carbinol (MIBC), rhamnolipid can produce foams with significantly higher elasticity and stability [10]. Therefore, in this study, no further frother was applied to the flotation process due to the high foamability of RL. The following chemicals were purchased from Merck GmbH (Germany) in analytical grades: sodium dichromate dihydrate (Na_2_Cr_2_O_7_·2H_2_O) as a metal ion source and ferrous sulfate (FeSO_4_·7H_2_O) as a metal reductant. The solution pH was also regulated using HCl and NaOH.

### 2.2. Bioflotation Variables and Experiment

To evaluate the chromium removal from the aqueous solution using bioflotation, effects of the operating variables including the initial pH of the solution (2–12), the RL to metal ratio (RL/Cr, 0.01–0.1), aeration rate (50–250 mL/min) and the reductant to chromium ratio (Fe/Cr, 0.5–3) were assessed using statistical analyses. The studied operating variables have been selected with some modifications based on those studies relevant to chromium bio-removal as reported by references [16,17,18,19,20,21,22].

**Figure 1:**
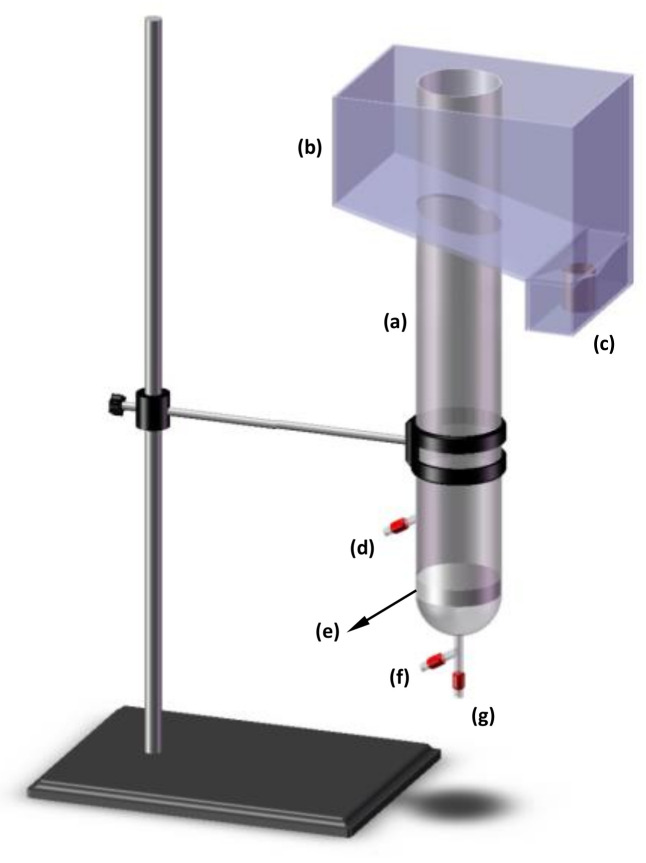
Schematic illustration of the used flotation column: (a) froth column, (b) froth box, (c) froth discharge gate, (d) sampling tube, (e) fritted glass sparger, (f) air inlet connected to rotameter, pressure gauge and air compressor and (g) discharge outlet for liquid diffused from sparger.

The bioflotation tests were carried out in an acrylic column cell with a height and diameter of 60 cm and 5.7 cm, respectively. The bubbly regime was generated at a carefully predetermined rate through a sintered glass frit (10–15 μm) from the bottom of the column. Figure 1 presents a schematic diagram of the experimental set-up. For every individual experiment, the column was first filled with 1 L of chromium contaminated solution of 50 ppm concentration. Then, a requisite amount of biosurfactant was added to the solution and pH was adjusted to the given level. The flotation was begun by introducing air at a predetermined flowrate from an air compressor with enough capacity to support bubble generation during the flotation course (5 min). The pregnant foam formed over the liquid column was drained through the froth launder (Figure 1a) into a collecting vessel. To pursue the kinetics behaviour of the bioflotation process, the solution was slowly sampled at appropriate sampling intervals via a thin sampling tube installed on the column. The experimental program was implemented at ambient temperature of 26 + 1 °C. Each sample was analyzed twice and an average value was reported. The concentration of Cr of each sample was measured using an atomic absorption instrument (Varian model SperctAA 220, Mulgrave, Victoria, Australia). The bio-flotation efficiency of chromium, *R*_Cr_, was calculated as below [33]:

(1) $$R_{\mathrm{Cr}},\%=\frac{\left(C_{0}-C_{t}\right)V_{\mathrm{wt}}}{C_{0}V_{w0}}\times 100$$

where *C*_0_ is the chromium concentration in the bulk solution, *C*_t_ is the incremental concentration at time *t* (seconds), *V*_w0_ is the volume of the bulk solution, and *V*_wt_ is the volume of solution that remained in the column after *t* (seconds).

### 2.3. ANN Simulation

Neural networks are invaluable tools used in numerous complex fields, including image processing, observations, segregation, pattern recognition and control systems [34,35,36,37]. This wide acceptance of ANN approaches over classical simulation techniques comes from their rapid but also simple processing nature as well the capability to learn from historical and/or available examples. As a biological system, they can learn from examples and can deal with nonlinear problems. 

After designing a neural network training section, finding the best weight and bias for the neural network training using optimization algorithms is necessary. Currently, various algorithms are available, which may suffer from many shortcomings; for example, the conventional optimization algorithms generally converge at slow rates and may lead to response overfitting. Moreover, such old-fashion algorithms select the initial weight vector randomly, and thus fall into the local minimum. Metaheuristic algorithms can overcome such disadvantages. By proper training the ANN codes, they can learn the way input and output data interact with one another using a network of the input, hidden and output layer(s). The input layer transfers input data to hidden layers without any processing. Hidden layer(s) multiplies them to their corresponding weight and adding a bias using the equation below [29,38]:

(2) $$y_{i}=\overset{n}{\underset{i=1}{\sum }}f\left(w_{\mathrm{ij}}x_{i}\right)+b_{j}$$

where *x* is the input, *y* is the output of the neuron, *f* is an activation function, which can appear as either nonlinear or linear, and *n* is the number of inputs to the neuron. In this equation, the weight of the connection between neurons (i.e., neurons *i* and *j*) is designated by *w*_ij_ and the bias caused by *j^th^* neuron is thought of with *b*_j_. There are several metaheuristic algorithms, each with its strengths and weaknesses. Generally speaking, the following stages were carried out to develop the ANN structure and predict the removal efficiency of chromium bioflotation in the presence of RL biosurfactant:

- Data selection and preprocessing
- Divide the samples into two sets: training and test
- Select modeling method, geometry, and optimization algorithms
- Develop appropriate ANN model and verification of results

In this study, cuckoo, genetic, firefly and biogeography-based optimization algorithms were used. Each of these algorithms as well as steps involved in their code development are described briefly as follows.

#### 2.3.1. Optimization Algorithms Applied

**Figure 2:**
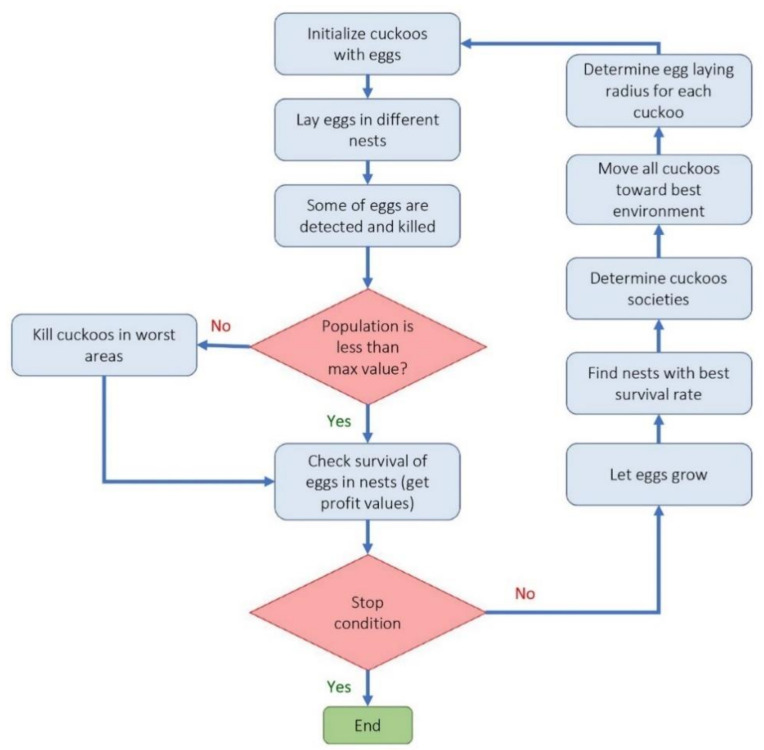
Flowchart of optimization process using cuckoo algorithm.

**Figure 3:**
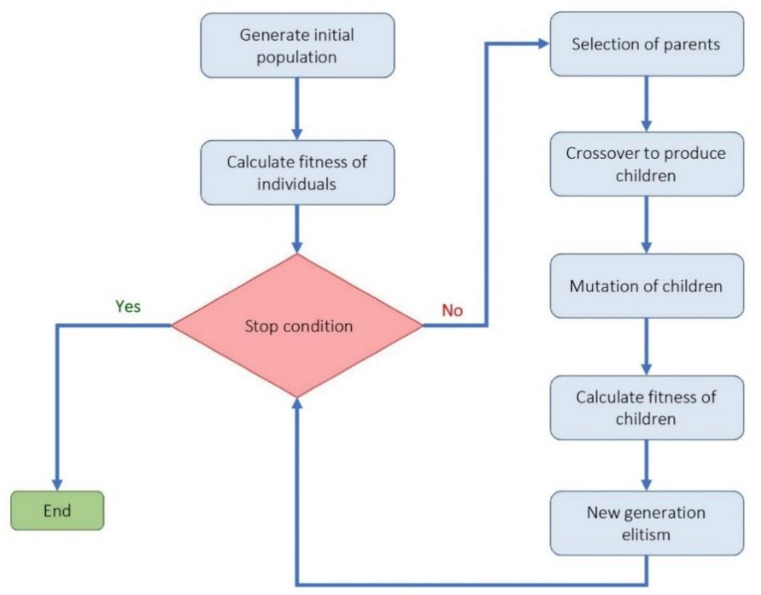
Steps involved in development of genetic optimization algorithm.

**Figure 4:**
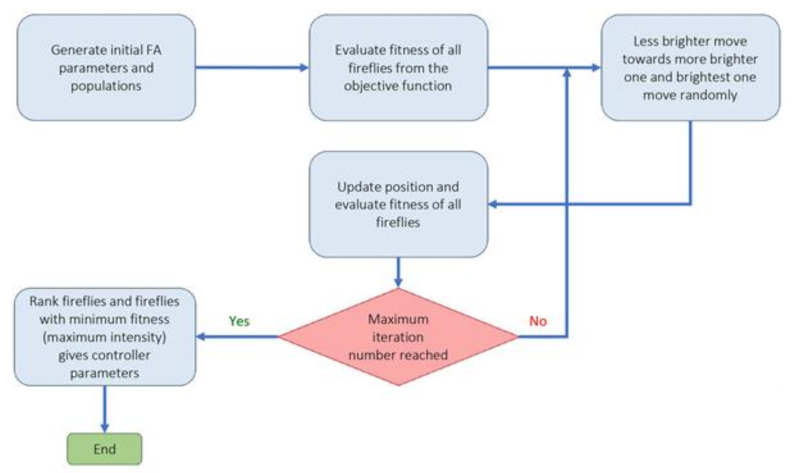
General instruction for problem solving by firefly optimization algorithm.

**Figure 5:**
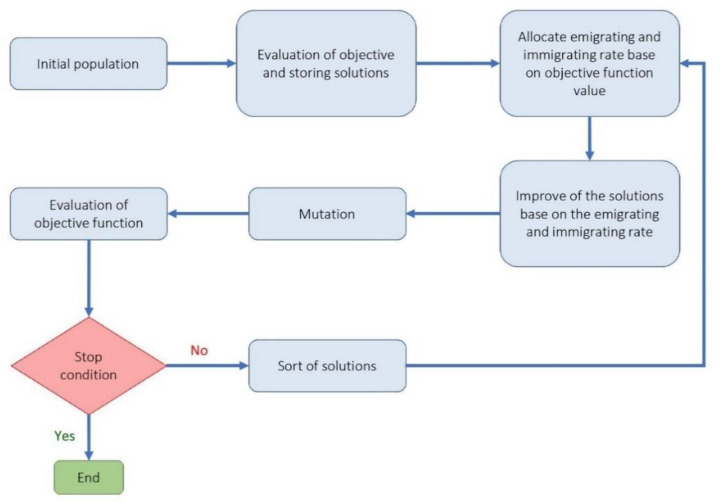
Flowchart of biogeography-based optimization algorithm.

(a) Cuckoo optimization algorithm (COA): Cuckoo optimization algorithm developed to solve nonlinear and/or continuous processes [39]. This algorithm is driven from the lives of a family of birds called cuckoo and is based on the optimal lifestyle and exciting features of this species, such as spawning and reproduction. Adult cuckoos and cuckoo eggs make up the initial population of the cuckoo optimization algorithm. Adult cuckoos lay their eggs in other birds’ nests. If the cuckoo eggs are not detected and destroyed by the host birds, they will grow into adult cuckoos [40]. Adult cuckoos migrate *en masse* under the influence of environmental characteristics and hope to find an optimal environment for life and reproduction. In this algorithm, the optimal environment will be the global optimum in the optimization problem’s objective function. This algorithm has so far performed well in various optimization scenarios and real-world applications [41,42]. Figure 2 illustrates the general route used for a COA development.
(b) Genetic optimization algorithm (GA): The genetic algorithm is a subset of computational models inspired by the concept of evolution. This algorithm encodes potential or candidate solutions for a particular problem in a chromosome-like data structure [43]. Implementation of a genetic algorithm usually begins with producing a population of chromosomes (the initial population of chromosomes in genetic algorithms is usually randomly generated and bound to the upper and lower limits of the problem variables). Next, the generated data structures (chromosomes) are evaluated, and chromosomes that better represent the problem’s optimal solution have a favorable reproduction chance than other chromosomes. In general, the goodness of an answer is usually measured concerning the population of obtained answers. Nowadays, due to this algorithm’s capabilities, especially in solving regression problems, it has a good position among other optimization methods [44,45]. The steps used to develop a genetic optimization code are shown in Figure 3.
(c) Firefly optimization algorithm (FFA): The firefly optimization algorithm is inspired by firefly behavior—they live together in large collections—and is one of the most efficient algorithms when solving hybrid optimization problems [46]. The firefly algorithm is a good example of collective intelligence in which agents that do not have very high abilities on their own can achieve great results by working together. The main assumptions of this algorithm are as follows [47,48]: (i) Fireflies are attracted to each other regardless of gender. (ii) The factor of attraction is proportional to their brightness; the brighter Firefly absorbs the lighter firefly. However, as the distance between the two fireflies increases, the attractiveness decreases. (iii) The fireflies with the same brightness move randomly. New pathways are created arbitrarily and generally lead to bright fireflies. Based on the flashing behavior of fireflies and the characteristics of their biological connections, Yang modeled firefly behaviors and developed this algorithm in 2010 [49]. Figure 4 illustrates a simple structure to optimize problems based on FFA.
(d) Biogeography-based optimization algorithm (BBO): The BBO algorithm, like the genetic and the firefly algorithm, is one of the collective intelligence algorithms. It is a nature-based method that uses the principles of biogeography to find the answer. In general, biogeography, as a sub-branch of biological science, studies different species’ behavior in different times and places [50]. In the BBO algorithm, each biological zone is recognized as a single member and has its habitat suitability index (HSI). In this algorithm, the answer or biological region with higher HSI indicates a better answer [51]. In BBO, properties are usually migrating from regions with higher HSI to regions with lower HIS. In other words, regions with low HSI take properties from regions with higher HSI. Each region’s variables are called suitability index variables (SIV), which express each region’s properties and are used in migrations. The BBO algorithm is developed by Simon to solve optimization problems and generate responses that maximize HIS [52]. The main steps to develop a genetic optimization code are shown in Figure 5.

#### 2.3.2. Data Preparation and Pre-Processing 

Due to the fact that the chief goal of the present reseaarch study is to simulate the process of bioflotation using expert systems, the number and conditions of experiments were determined such that there is no regular statistical relationship between them. For this purpose, a specified code in Matlab^®^ software (Mathworks R2018a v9.4, Natick, MA, USA) was developed to adjust the test conditions to: (i) each variable must appear at least once in the experimental design, (ii) each level of each variable must appear at least once in the experimental design, (iii) the experiments are randomly sorted in the final experimental design, (iv) each experiment should not be replicated more than twice and (v) replications should not include more than half of the total of the main experiments. The last two steps were defined in order to determine the error of statistical analysis and to prevent overlap (bias) of the main effects of the variables with each other. Finally, 113 individual experiments were defined and they were performed according to the experimental design created by the developed code.

**Figure 6:**
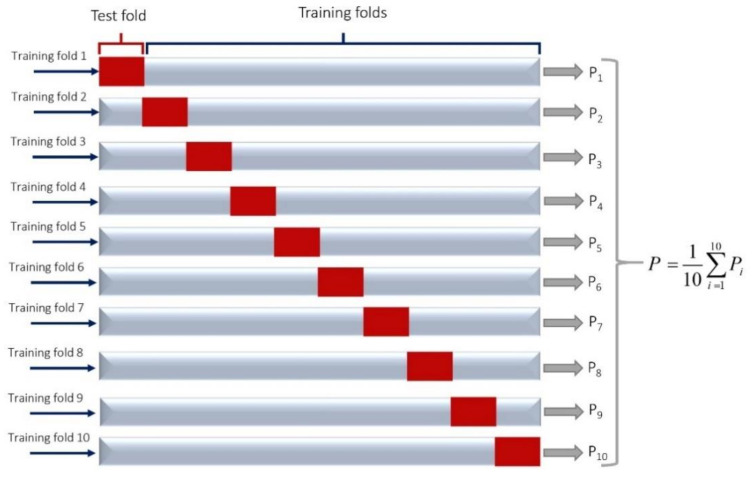
General structure of 10-fold cross-validation used in this study.

Normalizing is an essential step in data pre-processing and allows different dimensions to be fairly examined by the algorithm. For this reason and before feeding the data into the networks, they were normalized by the following equation:

(3) $$X_{c}=\frac{X_{i}-X_{min}}{X_{max}-X_{min}}$$

where *X_c_* and *X_i_* are normalized and actual values, respectively. *X_min_* and *X_max_* are the minimum and maximum value of each subset (inputs-outputs). Also, The K-fold cross-validation method is used in this study to reduce the over-fitting problem in model [53]. There are several cross-validation methods which have similar results; for instance, Badawy et al. [54] used the scanning test set to have a good representative training and test sample sets in their study. In the K-fold cross-validation method, the training dataset is divided into K sections at each level and model accuracy is calculated for K times. In this study, we used 10-fold cross-validation (Figure 6), and the percentage of training and test data were equal to 75% and 25% of the total data, respectively.

In this study, inputs are pH, RL/Cr ratio, air flowrate (mL/min) and Fe/Cr ratio. The output data is chromium removal (%) and four models were developed to predict it based on the input data. Table 2 lists the statistical levels of the data used in simulation studies.

## 3. Results and Discussions

### 3.1. Statistical Analysis of Experimental Results

**Figure 7:**
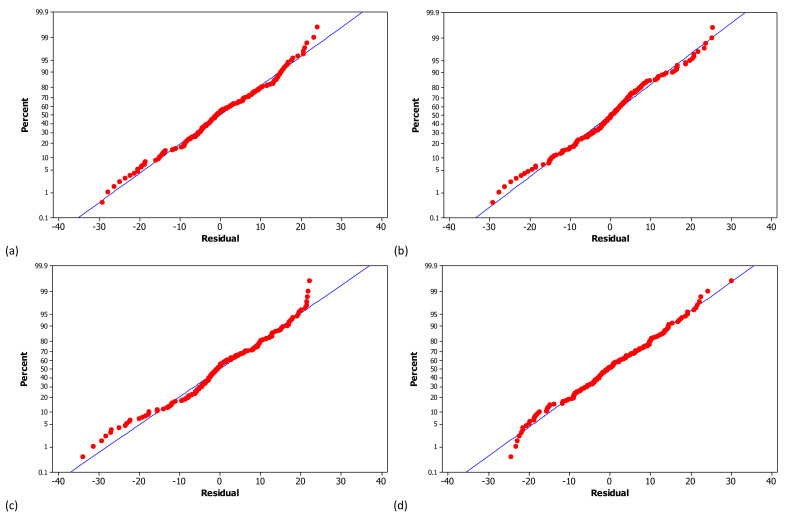
Normal probability plots of chromium removal versus (**a**) solution pH, (**b**) RL/Cr ratio, (**c**) aeration rate and (**d**) Fe/Cr ratio.

The effect of operating variables on chromium removal was assessed by running the one-way Analysis of Variance (ANOVA). Statistical assessments were conducted at 95% confidence level. However, the normality of process response relative to operating variables is a prerequisite to ANOVA. In this paper, the Anderson-Darling approach was considered as the normality analysis strategy that is commonly employed for engineering investigations [55]. In this method, the Anderson-Darling factor (AD) is defined as the measure of data normality as follows [56]:

(4) $$AD=n\int_{-\infty }^{\infty }\frac{{\left(F_{n}\left(x\right)-F\left(x\right)\right)}^{2}}{F\left(x\right)\left(1-F\left(x\right)\right)}dF\left(x\right)$$

where n is the number of individual data, F and F_n_ are the hypothesis and actual distribution functions, respectively. If AD value is less than unity and the corresponding *p*-value is over 0.05 (for 95% confidence interval), the data distribution follows a normal trend. The normal plots of chromium removal versus operating variables are shown in Figure 7 where the plotted points adequately formed a straight line and they are close to the normal distribution line. Comparing the normal plots and normality factors in Table 3 reveals that chromium removal follows normal trend relative to all variables because the corresponding *p*-values are greater than the confidence level (0.05) and AD-values are sufficiently low. The ANOVA results are also listed in Table 3 and clearly reveal that except for aeration rate, all other operating factors significantly influence the chromium removal as the *p*-value of all responses is obviously less than the significance level, i.e., 0.05 [57].

### 3.2. Effect of Solution pH

**Figure 8:**
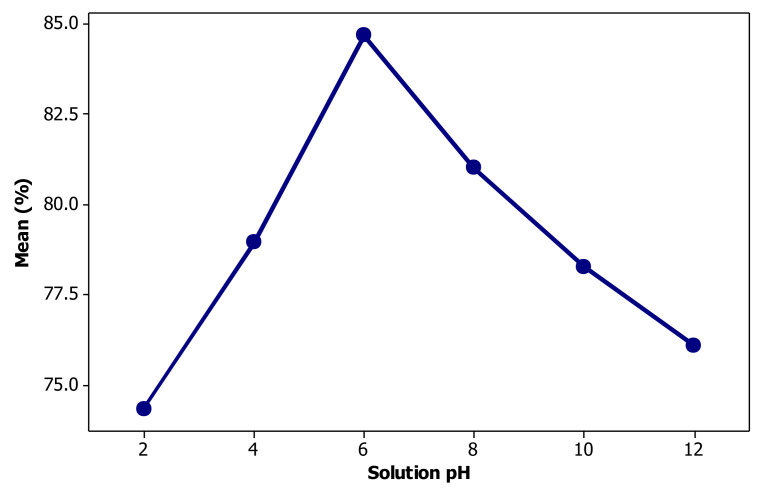
Effect of solution pH on the performance of chromium bio-flotation.

**Figure 9:**
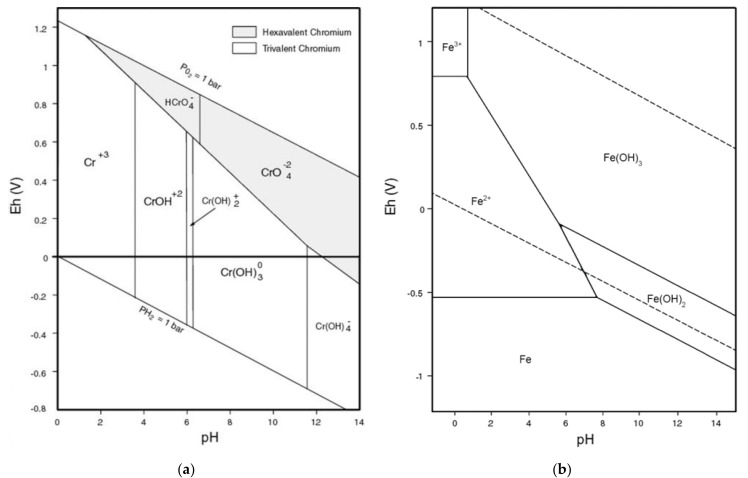
Chemical species of (**a**) chromium [61] and (**b**) iron [62] as a function of solution pH and potential.

The effect of solution pH on bio-flotation efficiency of chromium is given in Figure 8 showing that Cr removal increases by increasing the pH from 2 to 6 and then it significantly decreases. At pH 2 solely 74.33% of Cr can be removed from the solution while it reaches to 84.70% by increasing the pH to 6. Effect of solution pH can be explained from both Cr speciation in solution and the pH sensitivity of rhamnolipid activity. The pH of a solution is an important factor in flotation of metallic cations which determines the ion species in the solution and subsequently the way collector interacts with them. The Eh-pH diagram for chromium is shown in Figure 9. The starting metal source for bioflotation experiments was a hexavalent chromium compound. As solution pH decreases, HCrO_4_^–^ is the dominant component; however, the use of iron cation reduces the chromium valency from 6 to 3. Therefore, positively charged species of Cr(OH)_2_^+^ and CrOH^2+^ can interact with rhamnolipidic anions to improve the bioflotation performance. At intensive acidic conditions, instead of increasing positively charged spots in the solution, rhamnolipid biosurfactants lose their activity, which is discussed in the next paragraph. As solution pH exceeds neutral condition towards alkaline values, the interaction between rhamnilipidic anions and Cr species interruptions due to an attenuation of positive charge ions in the system. Another negative effect of alkaline conditions is related to deactivation of iron ions as hydroxide precipitates (Figure 9).

The surface activity of rhamnolipids is directly affected by the pH of the solution. The solution pH plays a key role in degree of molecular dissociation of rhamnolipids and controls the repulsion forces among them and consequently, the compaction degree of the monolayer of RL molecules adsorbed on the surface of air bubbles. Moreover, the elasticity of the liquid film at bubble surface is inversely influenced by the compressibility of the RL monolayer. It is well demonstrated that rhamnolipid biosurfactants present their maximum activity at pH values about 6–6.5 because the electrostatic repulsion forces between oxidryl head groups in the RL molecules increase [58]. As pH decreases down to about 4.5–5, RL molecules form monolayers with higher degree of compaction at the bubble surface. This phenomenon results in the formation of foam film with higher rigidity, lower elasticity and finally, less stable foam package which disrupts easily as air flow rate increases [32]. At highly acidic conditions, rhamnolipid molecules tend to precipitate as the carboxyl anions and lose their charge through bonding with hydrogenated functions [59]. The alkaline environment may increase the electrolytic effect among Na^+^ ion dissociated from sodium hydroxide (as pH regulator). The anionic character of rhamnolipid molecule is related to the presence of carboxyl group in its molecular structure. Electrolytes can force most of this anionic functions to dissociate in the form of carboxylate groups with negative charge. Therefore, addition of a mineral salt increases the ionic strength of the solution such that a diffuse layer of counterions will shield carboxylate groups the formerly dissociated in the solution [60]. 

### 3.3. Effect of Rhamnolipid Concentration 

**Figure 10:**
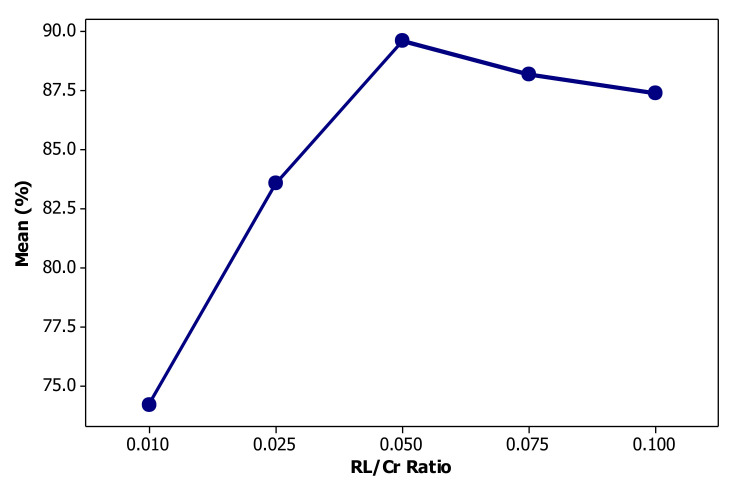
The role of rhamnolipid concentration in the performance of chromium removal by the bio-flotation process.

The impact of RL concentration on the efficiency of chromium bio-flotation is illustrated in Figure 10, indicating that as the RL dosage is increased, the efficiency of Cr bio-flotation follows an ascending trend up to a maximum value at the RL/Cr ratio of 0.05. The key role of RL in bio-flotation is as a Cr collector rather than a frothing agent. RL molecules adsorb on the bubble surface such that their hydrophobic hydrocarbon chains orient toward the air phase confined in bubbles while the carboxylate anions settle at the surface toward the aqueous solution. As the concentration of biosurfactant increases, more anions accumulate at the bubbles’ surface that, in turn, can gather more chromium ions outward the flotation column. From a foamability viewpoint, the excess concentration of RL may result in the formation of liquid film with undesirably enhanced elasticity at the surface of bubbles and finally, an over-stable foam [10]. 

High froth stability lowers bubble coalescence and consequently, diminishes drop-back of ions to the solution. As seen in Figure 10, the removal efficiency slightly decreases at high level of rhamnolipid concentrations (RL:Cr >0.050). Mass concentration values corresponding to the experimental levels of RL are 4 ppm for low, 24.4 ppm for mid and 44.4 ppm for high. Rhamnolipid biosurfactant can form micelle at concentrations around 10.1 ppm [30]. At concentrations over the critical micelle concentration (CMC), the hydrocarbon chains of RL molecules interlink via Van der Waals forces and do not adsorb at the air/water interfaces forming the surface of bubbles. Therefore, at RL/Cr ratios higher than 0.05, the carrying capacity of bubbles significantly decreases with regard to the lack of sufficient number of active, free RL molecules. Another negative consequence of excessive concentration of biosurfactant can be attributed to the potential competition between free RL anions and coligand-RL complex for a free space on the surface of air bubbles [63].

### 3.4. Effect of Aeration Rate

**Figure 11:**
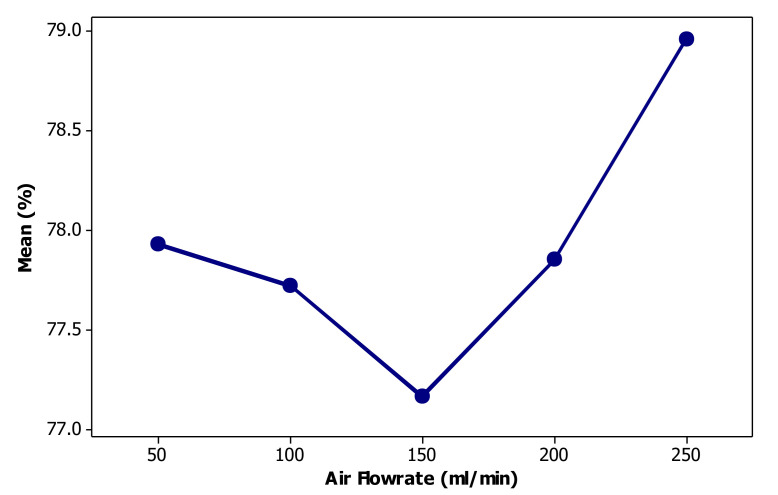
Effect of air flowrate on the performance of removing chromium through the bio-flotation process.

Figure 11 illustrates the effect of aeration rate on chromium bio-flotation performance. As shown, at an aeration rate of 50 mL/min, the Cr is removed 77.93%, which decreases with increasing aeration rate up to 150 mL/min and then increases sharply with increasing air flowrate. The maximum efficiency of 78.96% is obtained by applying an aeration rate of 250 mL/min. It is well established that in the flotation-related processes there is an optimum aeration rate for reaching the maximum separation efficiency [64,65]. During the experiments, it was observed that with increasing air flowrate, a very turbulent flow of large bubbles was formed in the column. This large bubbly regime created an intensive turbulent condition at the interface between the foam and the aqueous phase, causing the foam to become unstable. The bubbles proceeded to burst due to an increase in the coalescence rate, and finally the drain-back of the chromium loaded liquid to the column. By increasing the aeration rate, turbulence was reduced due to the increase of gas hold-up in the liquid phase. This led to improvement of foam stability and an increase in the removal of chromium. Also, increasing the aeration rate increased the water recovery (containing chromium species) and, consequently, the chromium removal efficiency. However, the effect of aeration rate on bio-flotation efficiency is not significant (Table 3) and removal efficiency varies by less than 2%.

### 3.5. Effect of Reductant Concentration

**Figure 12:**
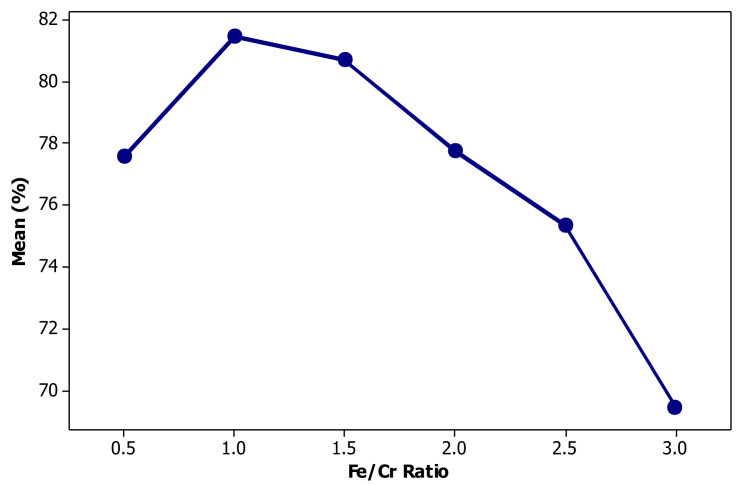
Effect of iron concentration on the performance of chromium bioflotation.

Figure 12 exhibits the effect of reductant concentration viz. Fe on chromium removal. As seen, the efficiency of chromium bio-flotation is initially increased as the Fe/Cr ratio was increased from 0.5 to 1 and then significantly drops from 1.5 to 3. The decrease of chromium removal at Fe:Cr ratios over 1.5 can be attributed to the likely competitive adsorption of chromium and iron cations by RL anions. At the ratio of less than unity, there is not sufficient iron cations to reduce anionic species of hexavalent chromium and thus, the removal efficiency reduces.

### 3.6. Interaction Effects and Process Optimization 

**Figure 13:**
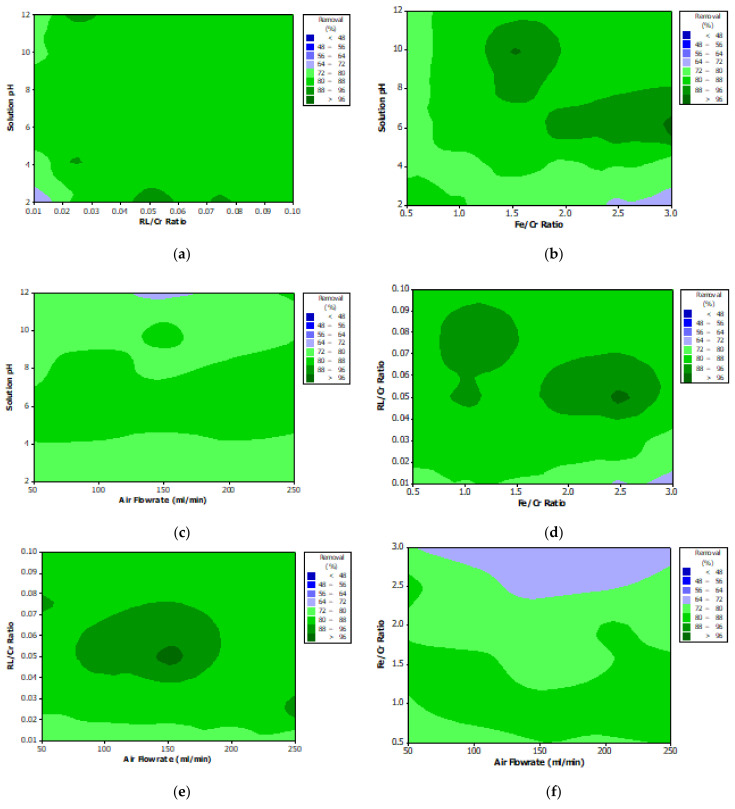
The interaction response plots between (**a**) pH and RL concentration, (**b**) pH and reductant concentration, (**c**) pH and air flowrate, (**d**) RL and reductant concentrations, (**e**) RL concentration and air flowrate and (**f**) reductant concentration and air flowrate.

The surface plots of the response of a process against other independent variables can provide useful information not only on the individual effect of operating factors but also on the potential interaction between them [66,67]. In this regard, the surface plots for the chromium bio-flotation efficiency were developed using experimental results. Figure 13 illustrates the surface response plots for interactions among four variables and shows that all interactions have non-linear effects on response. It is noteworthy that the interaction between RL/Cr and aeration rate (Figure 13e) shows that maximum removal can be obtained at mid-levels of aeration rates, whereas a contrary result was concluded from the individual effects. This conclusion can be directly attributed to the significant interaction between those variables. Other than studying the interactions, the surface plots are powerful tools for predicting optimal conditions resulting in maximum chromium removal. According to Figure 13, maximum bio-flotation efficiency can be achieved when all other operating variables vary close to the mid-level, i.e., pH of 6, rhamnolipid to metal ratio of 0.05, air flowrate of 150 mL/min, and reductant to chromium ratio of 1.0. However, to ensure the prediction accuracy, new tests were run under these conditions and chromium removal of 97.73 ± 0.13% was obtained.

### 3.7. Kinetics of Chromium Bioflotation

Flotation kinetics denotes variation of concentrated proportion of an entity, which floats as a function of time. Assessment of flotation kinetics is extremely helpful in understanding process mechanisms, and its outcomes can be considered a predictive indicator in relevant technologies [68]. To model and formulate its rate, researchers typically utilized chemical kinetics principles [69]. Various flotation kinetics modeling investigations showed that first-order kinetics equations and specifically classical flotation kinetics models perfectly present the process [70,71]. Thus, it was fitted to the experimental data to estimate the flotation rate constant and its maximum recovery (Equation (4)).

(5) $$R_{t}=R_{\infty }\left(1-e^{-\mathrm{kt}}\right)$$

where R_t_ (%) is the metal removal after t (s), R_∞_ (%) is the maximum removal achievable in practice, t (s) is the flotation time and k (1/s) is the kinetics rate. Fitting the experimental results with the model equation (5) revealed that kinetic characteristic of chromium bio-flotation is in a good agreement with the first-order classical flotation model with a correlation coefficient (R^2^) of 98.72%. The kinetics rate constant and R_∞_ were found to be 0.023 sec^−1^ and 97.54%, respectively.

## 4. Simulation Results

### 4.1. Artificial Neural Network Design

**Figure 14:**
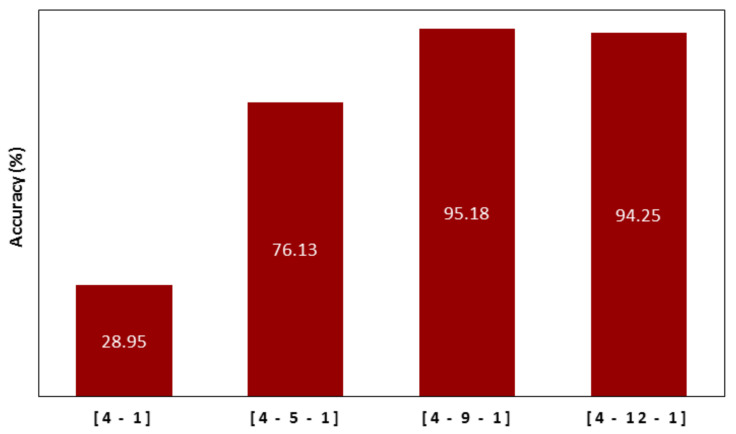
The overall accuracy of neural networks with different structures and number of neurons.

To model a dataset using ANN, underfitting and overfitting problems are common and must be prevented. A simple structure causes underfitting, while a highly complex one leads to overfitting. An overfitting model may be very accurate in the training phase but does not provide good results in the testing phase [30,72]. In this paper, the optimal structure of 4-9-1 was found for the neural networks with different optimization algorithms based on trial-and-error processes. In fact, according to the input data, various structures were tested for all the neural networks with the mentioned optimization algorithms, and the 4-9-1 structure showed the highest accuracy among them. This structure with an equal number of iterations and the sigmoid function was applied to each layer of ANNs. Figure 14 shows the general accuracy of the various structures. In ANNs, structures without hidden layers are required only if the data should be separated linearly. As shown in Figure 14, the structure without the hidden layer did not have acceptable accuracy, so the structures with the hidden layer were examined.

**Figure 15:**
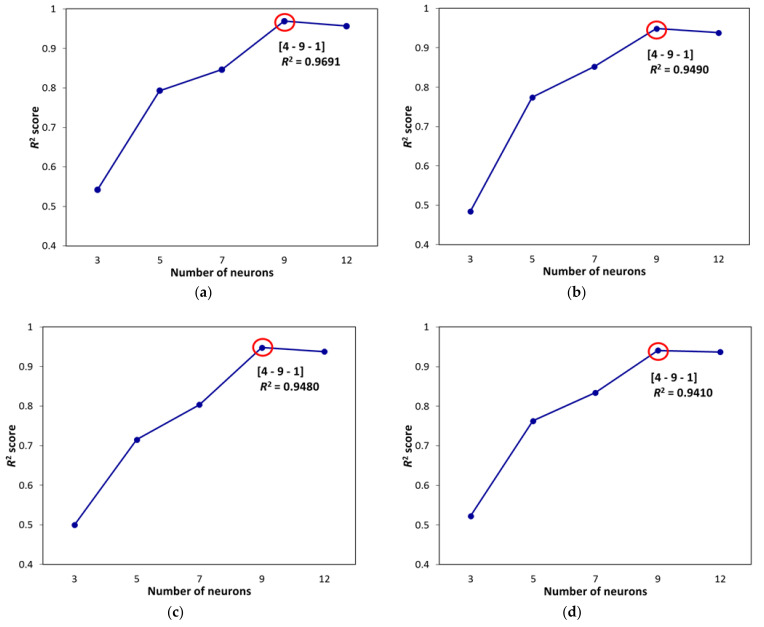
R^2^ score versus number of neurons in the hidden layer for (**a**) firefly algorithm, (**b**) genetic algorithm, (**c**) biogeography-based algorithm and (**d**) Cuckoo algorithm.

As mentioned earlier, the numbers of input and output variables in this work were 4 and 1, which are equal to the numbers of input and output neurons, respectively. Variation of R^2^ score with the number of neurons in the hidden layer is represented in Figure 15, which confirms nine neurons as a proper number of neurons in the hidden layer for all mentioned algorithms. In fact, besides a careful parameter selection for the optimization algorithms, to determine the appropriate structures, different structures were examined, and their results were compared with each other.

### 4.2. Evaluation of ANN Prediction Results

In this study, criteria for measuring neural networks accuracy and comparing their performance were the mean square error (MSE), root mean square error (RMSE), and percentage error. MSE and RMSE are presented in the following equations [73,74]:

(6) $$MSE=\frac{1}{n}\sum_{i=1}^{n}{\left(y_{i}-y_{i}\prime \right)}^{2}$$

(7) $$RMSE=\sqrt{\frac{1}{n}\sum_{i=1}^{n}{\left(y_{i}-y_{i}\prime \right)}^{2}}$$

where y and y’ represent the estimated and actual measured values, respectively, and n is the number of available data. 

**Figure 16:**
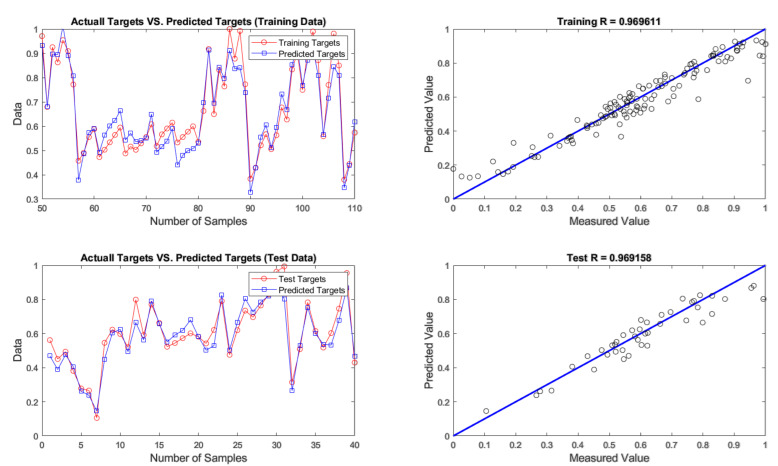
Results of training and testing data for predicting chromium removal using firefly algorithm.

**Figure 17:**
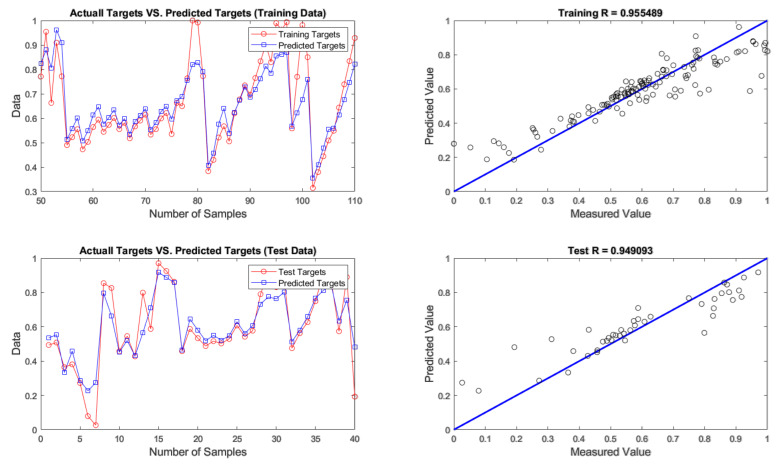
Results of training and testing data for predicting chromium removal using genetic algorithm.

**Figure 18:**
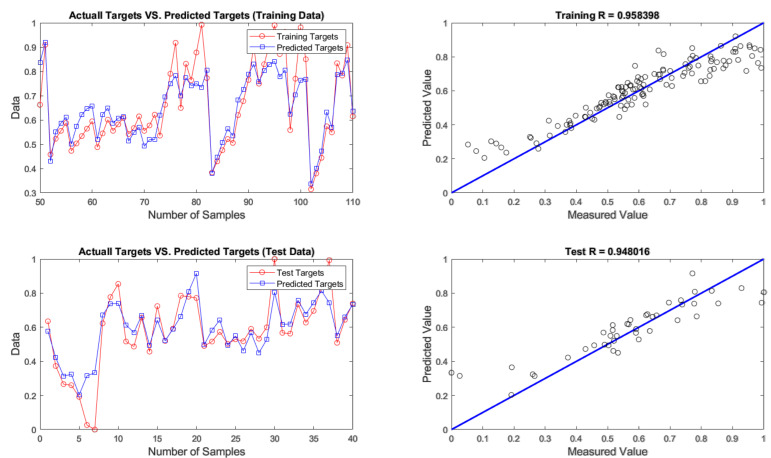
Results of training and testing data for predicting chromium removal using biogeography-based algorithm.

**Figure 19:**
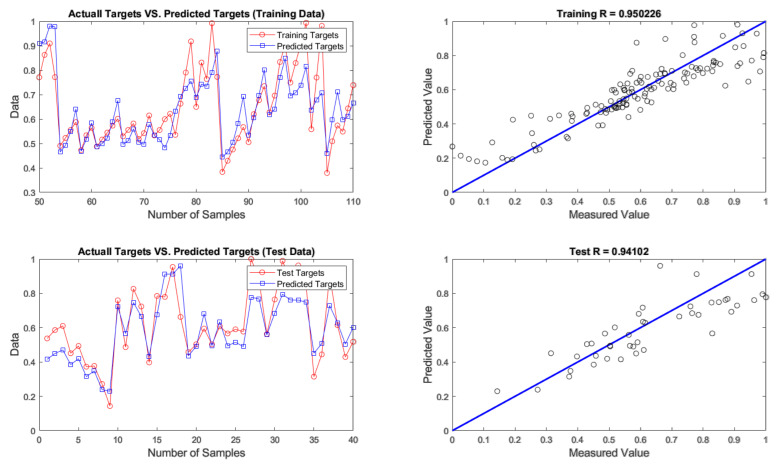
Results of training and testing data for predicting chromium removal using cuckoo algorithm.

After developing networks, the performance results of ANNs with different optimization algorithms were achieved. According to the results given in Table 4, firefly was found to be the best algorithm for predicting chromium removal response with 4-9-1 structure. In terms of predictive accuracy, after the firefly algorithm, the genetic algorithm is in the second place. The models’ prediction results are given in Table 4, fitting and regression diagrams of both the training and test results are shown in Figure 16, Figure 17, Figure 18 and Figure 19. It is important to know that the field of meta-heuristic algorithms is very experimental, and one algorithm’s performance depends on the type and structure of the problem. Thus, a very successful algorithm in one problem may have a poor performance in another and before experimental results, it cannot be stated with certainty that one algorithm is superior to another.

According to Table 4 and Figure 15, Figure 16, Figure 17 and Figure 18, the firefly algorithm provides the best accuracy to the neural network. This better performance of simulation is due to the fact that, to implement the algorithm and calculate the distance between two fireflies, the firefly algorithm is not limited to using the Euclidean distance like some of the mentioned methods, and any desired optimized equation can be used depending on the type of optimization problem [75]. The superiority of the firefly algorithm over the other algorithms studied here is that in addition to finding the global optimum, it can simultaneously find the local optimum of the optimization problems effectively [76]. One of the advantages that distinguishes the firefly algorithm from other conventional optimization algorithms is that different fireflies operate almost independently [77]. Such an important feature also makes the firefly algorithm an ideal choice for “parallel implementations” of evolutionary algorithms. Comparing the firefly algorithm’s performance with other optimization algorithms shows that this algorithm can converge to the global optimization of functions better and more efficiently. This algorithm, along with the neural network with an error rate of 3.08 percent, had the lowest error in predicting chromium removal in our problem. And the genetics, biogeography-based and cuckoo algorithms with error rates of 5.09, 5.20 and 5.90 percentage were the next best, respectively.

## 5. Conclusions

This paper examined the role of four effective factors including initial solution pH, rhamnolipid to chromium ratio, concentration of rhamnolipid, reductant (Fe) to chromium ratio, and air flowrate in removing chromium from an effluent. The process was optimized through four advanced optimization techniques based on ANN including Cuckoo, genetic, firefly and biogeography-based algorithms with a structure of 4-9-1. Rhamnolipid was produced and then used as an environmentally friendly collector with reasonable froth properties for the chromium ion flotation experiments. 

ANOVA results confirmed that all four studied factors except aeration rate have a statistically significant impact on the process. Anderson-Darlin analysis showed that the variables all followed the normal distribution function with *p*-values higher than 0.05 and reasonably low AD-values. According to the experimental results, 84.70% of Cr was removed at pH 6 where at acidic and alkaline ranges, the efficiency was dropped dramatically due to a reduction of positively charged ions in the solution and instability of biosurfactant. An RL:Cr ratio of 0.05 was chosen for the process in terms of providing an acceptable amount of collector dosage and reasonable degree of frothability. It was disclosed that by increasing the aeration rate from 50 to 150 mL/min, the Cr removal was reduced from 77.93% to 77.16%, while the maximum level of 78.96% was achieved by injecting air flow rate of 250 mL/min regarding an increase in the water recovery. The F:Cr ratio of 1.0 was proposed for the process because at the greater values a very competitive adsorption of Cr and Fe cations with rhamnolipid anions took place, which reduced the process efficiency substantially. Ion flotation kinetics studies revealed that the chromium bio-flotation follows the first-order kinetic rate with rate constant and ultimate recovery of 0.023 sec^−1^ and 97.54%, respectively. Moreover, the optimization results showed that the chromium removal by rhamnolipidic bioflotation could accurately be predicted using the firefly algorithm (FFA) with a properly selected structure of 4-9-1. In different stages of the ANN modeling, the acceptable correlation coefficients of 97% for both the training and testing outputs were achieved.

Last but not least, the current study may need further investigations from an experimental perspective, such as assessment of metal removal in the competitive multi-ionic system as well as simulation viewpoint, like evaluation of other optimization algorithms. A survey into the economic aspects of the process may also open up more horizons to its application at larger scales. Further studies are also required to clarify the kinetics, first principal modeling and scale up procedures in detail from different perspectives.

## Figures and Tables

**Table 1:** The ingredients of the modified medium used for bacterial cultivation.

Chemical Component	MgCl_2_	K_2_SO_4_	KH_2_PO_4_	Na_2_HPO_4_	NaNO_3_	MgSO_4_·H_2_O	CaCl_2_·2H_2_O	Agar
Dosage (g/L)	1.4	10	0.7	0.9	2	0.4	0.1	20

**Table 2:** The statistical summary of the data used in ANN simulation.

Parameter	Minimum	Mean Value	Maximum	Standard Deviation
pH	2	4.53	12	2.9153
RL/Cr ratio	0.01	0.02	0.1	0.0137
Air flowrate (mL/min)	50	131.76	250	71.4793
Fe/Cr ratio	0.5	1.40	3	0.8414
Chromium removal (%)	45.10	77.89	99.96	11.9887

**Table 3:** Results of normality test and analysis of variance of chromium removal against operating variables.

Analysis	Normality Analysis			ANOVA				
Measures	*AD*	*p*-value	Status	SS	MS	F Value	*p*-value	Status
Solution pH	0.504	0.402	Normal	2315	463	3.46	0.005	Significant
RL:Cr ratio	0.459	0.437	Normal	4591	1148	9.61	0.000	Significant
Aeration rate	0.391	0.163	Normal	46	11	0.08	0.989	Insignificant
Fe:Cr ratio	0.412	0.420	Normal	1941	388	2.85	0.017	Significant

**Table 4:** Evaluating results for the prediction of chromium removal.

Algorithm	Network Structure	Training			Test		
		MSE	RMSE	% Error	MSE	RMSE	% Error
FFA	4 - 9 - 1	0.0037	0.0608	3.0389	0.0038	0.0617	3.0842
GA	4 - 9 - 1	0.0079	0.0890	4.4511	0.0104	0.1018	5.0907
BBO	4 - 9 - 1	0.0069	0.0832	4.1602	0.0108	0.1040	5.1984
COA	4 - 9 - 1	0.0099	0.0995	4.9774	0.0139	0.1180	5.8980

## Data Availability

The data presented in this study is available on request from the corresponding authors. The data is not publicly available due to the confidentiality of the research work.
